# IRE1α undergoes a dynamic filament assembly cycle governed by unfolded proteins and BiP

**DOI:** 10.64898/2026.10.19.760176

**Published:** 2026-10-20

**Authors:** Melissa Méndez-Moran, Maxence Le-Vasseur, Daniel N. Itzhak, Wiktoria Sadowska, G. Elif Karagöz, Peter Walter, David A. Agard

## Abstract

The endoplasmic reticulum is a major hub of protein synthesis, modification and trafficking that relies on the Unfolded Protein Response (UPR) to balance its folding capacity. IRE1, the most conserved of the UPR sensors, undergoes a change in oligomeric state upon activation that propagates the stress signal from the ER lumen to its cytoplasmic kinase/RNase domains. Here we show that opposing inputs from unfolded proteins and the ER chaperone machinery govern a dynamic cycle of IRE1 assembly. A *bona fide* unfolded ER client directly binds the IRE1 lumenal domain (LD) and promotes higher-order oligomerization. Model peptides further reveal that full assembly into LD filaments depends on ligand length, sequence, and IRE1’s oligomerization interfaces. The ER Hsp70, BiP, counterbalances this process through two distinct mechanisms: i) an ATP-independent interaction via BiP’s nucleotide-binding domain buffers early IRE1 assembly by engaging its oligomerization interface, whereas ii) ATP-regulated BiP/ERDJ machinery actively dismantles assembled filaments. Together, our findings reconcile direct unfolded protein recognition with BiP-mediated regulation to establish LD oligomerization as a point of integration through which unfolded protein load and chaperone availability dynamically regulate IRE1 assembly state and signalling.

